# Effect of negative pressure wound therapy after surgical removal of deep-seated high-malignant soft tissue sarcomas of the extremities and trunk wall—study protocol for a randomized controlled trial

**DOI:** 10.1186/s13063-022-06468-6

**Published:** 2022-06-18

**Authors:** Müjgan Yilmaz, Andrea Thorn, Michala Skovlund Sørensen, Claus Lindkær Jensen, Michael Mørk Petersen

**Affiliations:** Department of Orthopedic Surgery, University Hospital of Copenhagen, Rigshospitalet, Inge Lehmanns Vej 6, 2100 Copenhagen, Denmark

**Keywords:** Sarcoma, Soft tissue sarcoma, Negative pressure wound therapy, Prevena™, Extremity, Trunk wall

## Abstract

**Background:**

Sarcomas are a heterogeneous group of rare malignant tumors of mesenchymal origin in the musculoskeletal system. The main treatment is surgery often supplemented with pre-or postoperative radiotherapy.

A retrospective study by Bedi et al. indicated that negative pressure wound therapy (NPWT) reduced the risk of postoperative wound complications in patients treated with preoperative radiation followed by surgical tumor removal of lower extremity soft tissue sarcomas (STS), and the use of NPWT was not associated with an increased risk of local recurrence. Previous studies have shown that NPWT can reduce postoperative complications. STS surgeries are a high-risk procedure concerning wound complications.

**Methods:**

Non-blinded single-center randomized controlled trial comparing NPWT versus conventional wound dressing and postoperative wound complications after surgical removal of deep-seated high-malignant STS of the extremities or trunk wall

Sample-size calculation: 154 STS patients (80% risk of avoiding type II error, 5% risk of type I error, and an 80% wound complication risk)

Block randomization of 8 into:

Group A: Conventional wound dressing

Group B: NPWT (PREVENA PLUS™ Incision Management System)

Inclusion criteria: Surgery for a deep-seated STS of an extremity or the trunk wall

Exclusion criteria: Age < 18 years, plastic surgery, low malignant/borderline STS, chemotherapy, preoperative radiotherapy, allergic/hypersensitive to acrylic adhesives or silver, unwilling/unable to provide informed consent, metastatic disease, and ischemic surgeries

Primary study endpoints were set as major wound complications defined by O’Sullivan et al. as a secondary surgery under anesthesia for wound repairs and wound management without secondary surgery within 4 months postoperatively.

Secondary study endpoints among others are Musculoskeletal Tumor Society Score (MSTS), Toronto Extremity Salvage Score (TESS), and European Quality of Life - 5 Dimensions (EQ-5D).

Approval from the Scientific Ethical Committee and the Data Protection Agency has been obtained, and the study is registered at clinicaltrial.gov.

This study did not apply for external funding.

**Discussion:**

Many new medical devices and technical solutions are currently being introduced, and even though some documentation regarding the use of NPWT, e.g., in joint replacement surgery exist, it is also important to seek documentation for this treatment principle in STS surgery.

**Trial registration:**

Registered at ClinicalTrials.gov NCT04960332 and approved on 11 July 2021

## Background

Sarcomas are a heterogeneous group of rare malignant tumors of mesenchymal cell origin in the musculoskeletal system comprising 1% of all adult cancers [1]. The reported incidence of all types of sarcomas is approximately six to eight per 100,000 inhabitants corresponding to 300 cases per year in Denmark (250 Soft Tissue Sarcomas (STS) (100 retroperitoneal/abdominal STS), 50 bone sarcomas) [2, 3]. Sarcomas arise in the body’s connective tissues including bone, muscle, cartilage, fat tissue, blood vessels, and peripheral nerve sheaths and hence arise in all parts of the body, however, most dominant (78%) in the extremities [4].

There exist more than 50 histologic subtypes of STS yielding a broad heterogeneous morphology and biological behavior of all subtypes [4]. The incidence of STS increases in general with age (median age = 65) [4]. High-grade STS mostly metastasize hematogenous to the lungs, which is also the primary cause of sarcoma-specific death, and approximately 10% of patients with STS have metastases at diagnosis [5].

Treatment of STS requires a multidisciplinary highly specialized team, evaluating risks and benefits of all available options and expected outcomes with the aim to minimize the recurrence of disease and preserve function and quality of life. The main treatment principle that has largely been unchanged since the 1980s is surgery supplemented with radiotherapy depending on subtype and stage [6]. The use of negative pressure wound therapy (NPWT) after high-risk orthopedic surgeries such as amputations, treatment of open fractures, and joint replacement surgery are increasing, but the direct cost is much higher compared to the conventional wound dressing. However, previous studies have shown that NPWT can reduce postoperative complications such as wound dehiscence and infection [7–10]. STS surgery that is often combined with pre-or postoperative radiation therapy is a high-risk procedure concerning wound complications and postoperative infections [11–13]. A retrospective study showed that NPWT reduced the risk of wound complications in patients with lower extremity STS treated with preoperative radiation and the use of NPWT was not associated with an increased risk of local recurrence [14].

The purpose of this research project is to improve the surgical treatment of STS treatment based upon a randomized controlled trial (RCT). This study aims to compare standard wound closure with staples and conventional wound dressing (control group) with wound closure with staples and NPWT (interventions group). We want to evaluate the effect of the use of NPWT versus a conventional wound dressing on postoperative wound complications after surgical removal of deep-seated high-malignant STS of the extremities or trunk wall.

## Methods/design

### Study design

The study design is as follows: non-blinded single-center randomized (1:1) controlled trial, with parallel groups and block randomization of 8 patients in each group comparing NPWT versus conventional wound dressing and postoperative wound complications after surgical removal of deep-seated high-malignant STS of the extremities or trunk wall.

### Enrolment

Patients evaluated at a multidisciplinary conference and afterward with a biopsy verified STS will be identified in Sundhedsplatformen (electronic patient records); the only information needed from the patient records prior to informed consent is when they will meet in the outpatient clinic. Patients eligible for inclusion will at their first outpatient visit to plan further final surgery be informed about the study written and orally. On the day of their surgery, written informed consent will be obtained if they want to participate in this study. No patient will have below 48 h to answer, complying with the Helsinki declaration [15].

All patients will be informed orally and written by the principal investigator, or colleague information will be given in a separate room allocated for only the patient and if wanted the assessor, so interruption does not occur. All patients will be asked if they want an assessor, and then a new meeting will be arranged, and the information will again be given by the principal investigator in a separate room allocated for the patient and the assessor.

The written informed consent gives the principal investigator, sponsor, and sponsor’s representatives as well as any control authority direct access to obtain information in the patient’s medical record, etc., including electronic medical record, in order to see information about the subject’s health, which is necessary for carrying out the research project and for control purposes, including self-monitoring and quality control, which they are required to perform.

**Fig. 1:**
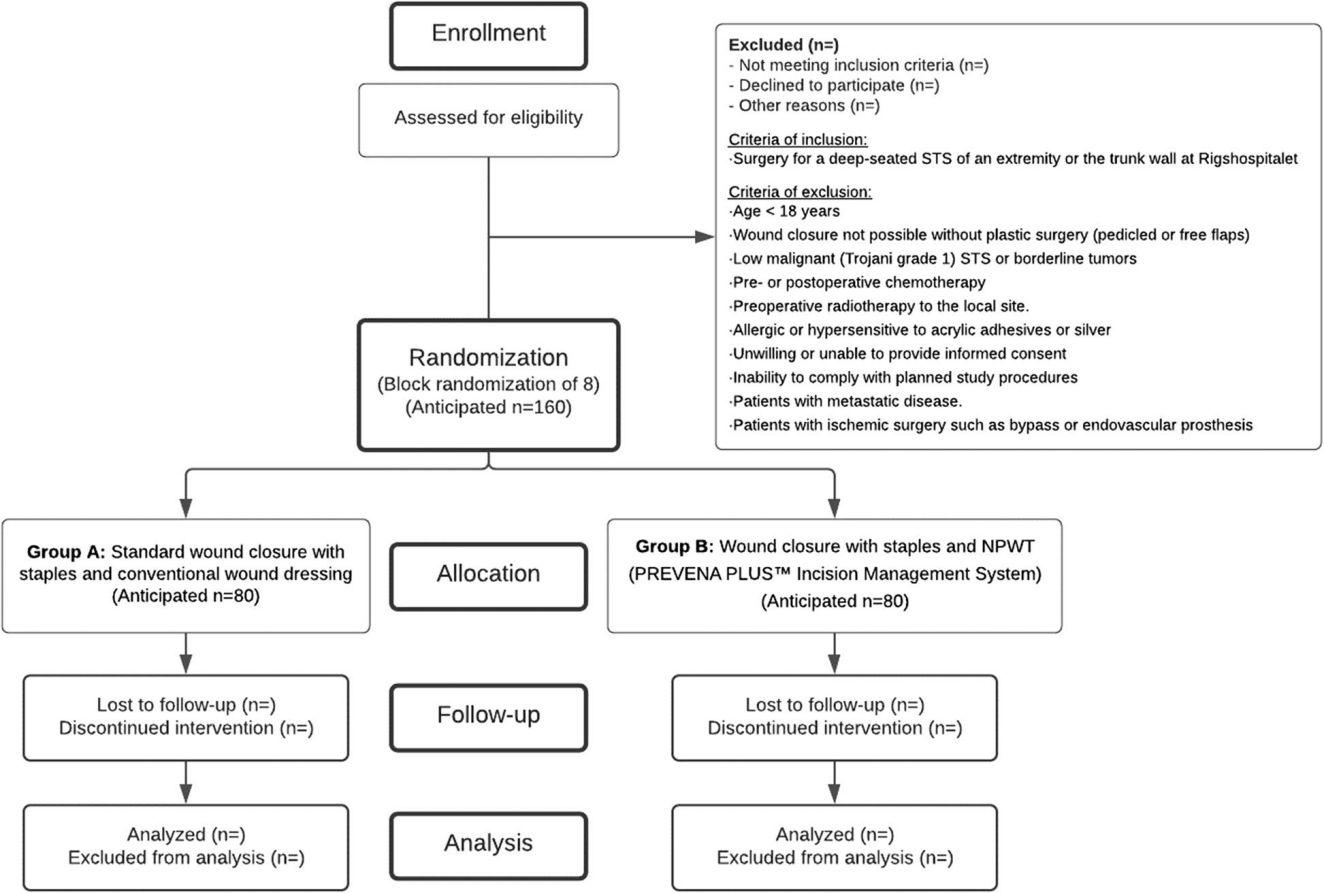
CONSORT expected flow diagram

The participants are covered by the Danish Patient Compensation Association (Fig. 1).

### Criteria of inclusion

- Surgery for a deep-seated STS of an extremity or the trunk wall at Rigshospitalet

### Criteria of exclusion

- Age < 18 years
- Wound closure not possible without plastic surgery (pedicled or free flaps)
- Low malignant (Trojani grade 1) [16] STS or borderline tumors
- Pre- or postoperative chemotherapy
- Preoperative radiotherapy to the local site
- Allergic or hypersensitive to acrylic adhesives or silver
- Unwilling or unable to provide informed consent
- Inability to comply with planned study procedures
- Patients with metastatic disease
- Patients with ischemic surgery such as bypass or endovascular prosthesis

### Drop out criteria

- Did not want to participate

### Variables collected

- Age
- Gender
- Lifestyle factors such as alcohol consumption, caffeine intake, smoking habits, BMI, and exercise
- Comorbidities, including medical treatments (including but not limited to hypertension, hypercholesterolemia diabetes mellitus/HbA1C, dialysis dependence, chronic venous insufficiency, end-stage renal disease, congestive heart failure)
- Radiation regime
- Operation time
- Blood loss at surgery
- Pathology
- Tumor location, size, grade depth
- Postoperative medical treatment
- Images

### Randomization

All 160 patients will in the operating room be randomized (randomization with 80 patients in each group) to receive one of the two treatments:

Group A: Standard wound closure with staples and conventional wound dressing

Group B: Standard wound closure with staples and NPWT (PREVENA PLUS™ Incision Management System)

Block randomization with 8 patients in each block stratified for upper extremity/truncal wall or lower extremity STS will be performed using a verified computerized irreversible application—the Research Electronic Data Capture (REDCap). The randomization sequence will be computer-generated. This study is not blinded.

The study takes place at the Department of Orthopedic Surgery, Rigshospitalet, University of Copenhagen. Patients will be followed in our outpatient clinic; the surgeries will be performed by specialized orthopedic tumor surgeons. Rigshospitalet is a tertiary multidisciplinary sarcoma center performing highly specialized tumor surgeries.

### Follow-up

The patient’s wounds will be followed by a member of the department staff or research group with photo documentation on day 0, day 7, at definitive wound healing (removal of staples), 4 months postoperatively, and in case of major wound complication.

Follow-up from day 7 and further on will be performed in the outpatient regime, at Rigshospitalet, orthopedic department; patients will be convened through e-books (secure platform for digital postboxes), and, if necessary, transport will be provided.

**Fig. 2:**
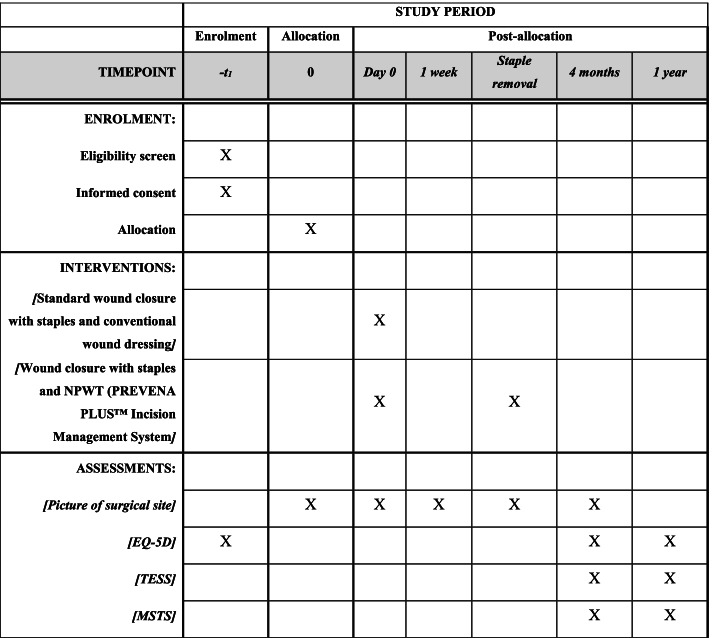
SPIRIT figure. Schedule of enrolment, allocation, interventions, and assessments of the 160 STS patients

Preoperative, after 4 months, and 1 year quality of life will be evaluated with European Quality of Life - 5 Dimensions (EQ-5D) [17]; furthermore, Musculoskeletal Tumor Society Score (MSTS) [18] and Toronto Extremity Salvage Score (TESS) [19] will be used to evaluate function 4 months and 1 year postoperatively depending on surgery location (Fig. 2).

### Patient involvement

There has not been any public or patient involvement when designing this protocol.

### Main hypotheses

The use of NPWT will result in a decrease of postoperative wound complications after surgical removal of deep-seated high-malignant STS of the extremities or trunk wall compared to a conventional wound dressing.

### Primary study endpoints

A major wound complication defined as in O’Sullivan et al. [11]:

- A secondary surgery under anesthesia for wound repairs such as debridement, operative drainage, and secondary wound closure
- Wound management without secondary surgery; this includes invasive procedure without anesthesia such as aspiration of seroma and readmission for wound care such as intravenous antibiotic or persistent deep packing within 4 months (120 days) after surgery

### Secondary study endpoints

Length of hospital stay, readmission to hospital for treatment of a wound complication, time to primary wound healing and removal of staples (ready for radiation therapy), deep infection, postoperative seroma development, MSTS [18], and TESS [19] 4 months and 1 year postoperatively; furthermore, quality of life will be evaluated with EQ-5D [17] preoperatively, at 4 months and 1 year.

### Statistical analysis

Fisher’s exact test will be used for categorical variables.

Depending on if the data could be considered normally distributed or not, a *t*-test for unpaired data or Mann–Whitney *U* test will be used for continuous variables.

Kaplan-Meier survival analysis will be used for overall survival (with log-rank test for comparison of groups). In case of an assumption of a difference in time to delayed wound healing, competing risk analysis will be used to address differences between the groups with death and amputation as competing factors.

All analyses will be performed in accordance with the intention-to-treat principles.

Two-sided *p*-values below 0.05 will be considered statistically significant.

### Calculation of sample size

In the study of O’Sullivan et al. [11], a major wound complication within 4 months postoperatively was identified in 16 out of 94 (17%) STS patients treated with surgery and postoperative radiation therapy. There exist no studies evaluating the effect of NPWT on wound complications after surgery for STS combined with postoperative radiation therapy. However, in retrospective studies, it was found that NPWT reduced the risk of wound complications from 47 to 8% (83% reduction) in patients with lower extremity STS treated with preoperative radiation [14] and from 27 to 7% (75% reduction) in patients undergoing hip and knee revision surgery [20].

In a study design with an 80% risk of avoiding type II error, a 5% risk of type I error, and an 80% wound complication risk reduction in a population similar to the study by O’Sullivan et al. [11], we will need to include 154 STS patients (77 in each group) in an RCT.

To make allowance for dropouts (since the follow-up period regarding the primary study endpoint is only 4 months = 120 days few dropouts are expected) during the study period, we plan to include 160 patients.

## Discussion

The surgical intervention in both groups is equal and according to current standards; the only difference is the wound dressing.

The randomization is planned as block randomization with 8 in each block; furthermore, a stratification of over extremity/trunk wall and under extremities will be performed; this is due to minimizing the risk of selection bias.

### Risks, side effects, and disadvantages

We do not expect that the patients participating in the study will experience any special side effects or complications related directly to the specific use of NPWT or the standard wound closure. The dressing may for some patients give a mild skin irritation of the skin under the enclosed dressing or a slight discomfort with having to wear an occlusive dressing. Any transient skin irritation (i.e., resolves within 24 h following device use and requires no medical intervention) will not be classified as an adverse device effect. Some blistering formations have been reported in relation to total hip and knee replacement surgery but no more than patients with conventional dressing [7].

A retrospective study showed that NPWT reduced the risk of wound complications in patients with lower extremity STS treated with preoperative radiation, and the use of NPWT was not associated with an increased risk of local recurrence [14].

No risk or side effects are reported by the manufacturer when adhering to the user manual.

Prevena™ NPWT is used daily at the orthopedic department; it is a CE-classified and FDA-approved medical device, and due to use in the recommended field, approval from the Danish Medicines Agency is not necessary.

### Ethical considerations

The study is approved by the Scientific Ethical Committee of the Capital Region of Denmark (H-21013549) and the Danish Data Protection Agency (P-2021-150), and it is registered at clinicaltrials.gov (NCT04960332) before the inclusion of patients. All patients will receive both oral and written information before written informed consent to participate is obtained.

The regulation from data protection regulation and data protection agency will always be followed.

The significant use of the present study is new knowledge concerning the best way to treat a certain group of patients with deep-seated STS of an extremity or the trunk wall. Knowledge from our study will benefit society in general and optimize utilization of resources, in terms of the best treatment for future patients.

In case of serious adverse preliminary results, data will be analyzed, and if one of the treatments is found to cause significant critical problems compared to the other (*p* < 0.05), the study will be ended. The study is thus designed to minimize unnecessary risks to the patients.

## Trial status

Protocol version: 07.05.2021 Version 1.2

Recruitment: started

Anticipated completion of recruitment: December 2024

## Data Availability

All data will be kept in REDCap, but datasets used and/or analyzed during this current study will be available from the corresponding author on reasonable request.

## Abbreviations

NPWT: Negative pressure wound therapy
RCT: Randomized controlled trial
STS: Soft tissue sarcoma
REDCap: Research Electronic Data Capture
EQ-5D: European Quality of Life - 5 Dimensions
MSTS: Musculoskeletal Tumor Society Score
TESS: Toronto Extremity Salvage Score
